# Redo Aortic Root and Total Arch Replacement for a Giant Ascending and Arch Aneurysm After a Previous Double Mechanical Valve Replacement

**DOI:** 10.7759/cureus.113370

**Published:** 2026-07-25

**Authors:** Salma El Manir, Siham Bellouize, Nabil Saouti, Aniss Seghrouchni, Younes Moutakiallah

**Affiliations:** 1 Cardiac Surgery Department, Mohammed V Military Training Hospital, Rabat, MAR; 2 Cardiac Surgery Department, Radboud University Medical Center, Nijmegen, NLD

**Keywords:** aortic arch aneurysm, deep hypothermic circulatory arrest, redo aortic surgery, selective antegrade cerebral perfusion, total arch replacement

## Abstract

Redo replacement of the ascending aorta and aortic arch after previous double mechanical valve replacement is rare and technically challenging. Surgical re-entry is particularly hazardous in the presence of giant aneurysms because dense adhesions, distorted mediastinal anatomy, and prosthetic valves increase the risks of complications. We report the successful management of a giant compressive aneurysm using a staged strategy incorporating pre-sternotomy left ventricular (LV) apical decompression. A 63-year-old man with previous mitral commissurotomy and double mechanical mitral-aortic valve replacement presented with progressive dysphagia and severe dyspnea. Computed tomography revealed a giant ascending and arch aortic aneurysm (117 × 116 mm) causing marked tracheoesophageal compression. Given the high risk associated with redo sternotomy, a staged surgical strategy incorporating pre-sternotomy LV apical decompression through a left anterolateral thoracotomy, systemic cooling, and selective antegrade cerebral perfusion was employed. Redo aortic root and total arch replacement was successfully performed with a 24-mm quadrifurcated graft and reimplantation of the supra-aortic trunks and coronary buttons. Despite a complicated postoperative course, the patient recovered fully, and four-month follow-up imaging confirmed graft integrity and resolution of compressive symptoms. This case demonstrates that giant ascending and arch aneurysms occurring after previous double mechanical valve replacement can be successfully managed using a carefully staged reoperative strategy. Pre-sternotomy LV apical decompression may represent a valuable adjunct for safe re-entry in selected patients with large sternal-adherent aneurysms.

## Introduction

Giant aneurysms of the ascending aorta and aortic arch are uncommon but may cause life-threatening complications, including rupture and compression of adjacent mediastinal structures [1]. Their surgical management is particularly challenging in patients undergoing redo cardiac surgery because prior sternotomy, mediastinal adhesions, and altered anatomy increase the risks of catastrophic injury during re-entry [2].

Safe re-entry and organ protection are therefore critical considerations in complex redo aortic procedures. We report the successful management of a giant compressive ascending and arch aortic aneurysm occurring decades after commissurotomy and double mechanical valve replacement performed using 23-mm aortic and 29-mm mitral St. Jude Medical (SJM) mechanical prostheses (Abbott Laboratories, Abbott Park, IL, USA), highlighting a staged re-entry strategy incorporating pre-sternotomy left ventricular (LV) apical decompression, controlled systemic cooling, and redo aortic root and total arch replacement.

## Case presentation

A 63-year-old man with a history of rheumatic heart disease underwent closed mitral commissurotomy in 1986 for severe rheumatic mitral stenosis, followed by double mechanical valve replacement in 1991 with 23-mm aortic and 29-mm mitral SJM mechanical prostheses. He presented with progressive dysphagia and New York Heart Association (NYHA) class IV dyspnea.

**Figure 1:**
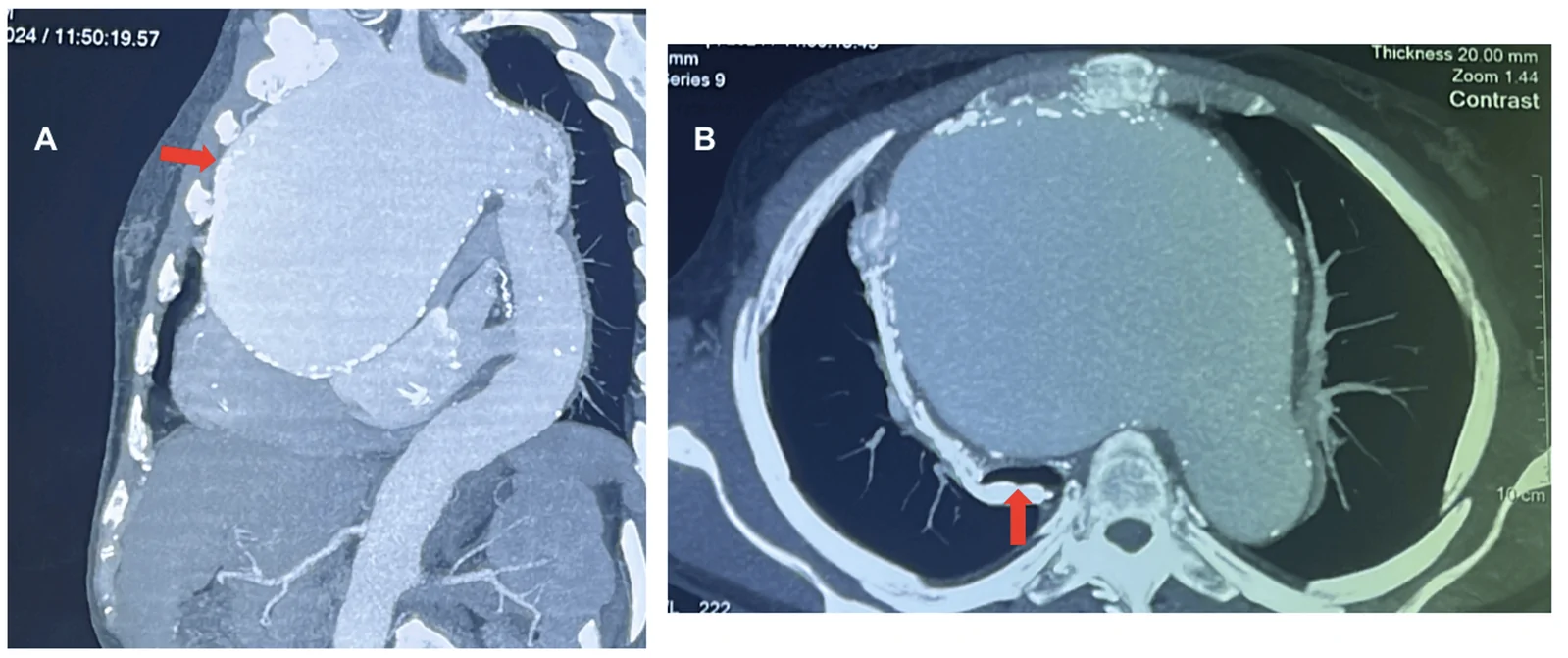
Preoperative contrast-enhanced computed tomography (CECT) (A) Sagittal reconstruction showing a giant ascending and arch aortic aneurysm (red arrow). (B) Axial section demonstrating tracheal compression and deviation (red arrow) by the aneurysm.

Contrast-enhanced computed tomography (CECT) revealed a giant ascending aortic aneurysm measuring 117 × 116 mm with extension into the aortic arch, resulting in marked tracheal and esophageal compression (Figure 1).

Transthoracic echocardiography revealed severe tricuspid regurgitation secondary to extrinsic compression, while both mechanical prosthetic valves were functioning normally with preserved biventricular function.

Given the high risk associated with redo sternotomy and the giant compressive aneurysm, a staged operative strategy was planned. First, femoro-femoral cardiopulmonary bypass was established via the right common femoral artery (19-Fr arterial cannula) and right common femoral vein (25-Fr two-stage venous cannula with vacuum-assisted venous drainage) to initiate controlled systemic cooling.

Through a left anterolateral thoracotomy, an LV apical vent was inserted to achieve early cardiac decompression and prevent ventricular distension in the event of fibrillation. After cooling to 30°C, median resternotomy was performed, followed by careful dissection of the aneurysm, supra-aortic trunks, and innominate vein.

**Figure 2:**
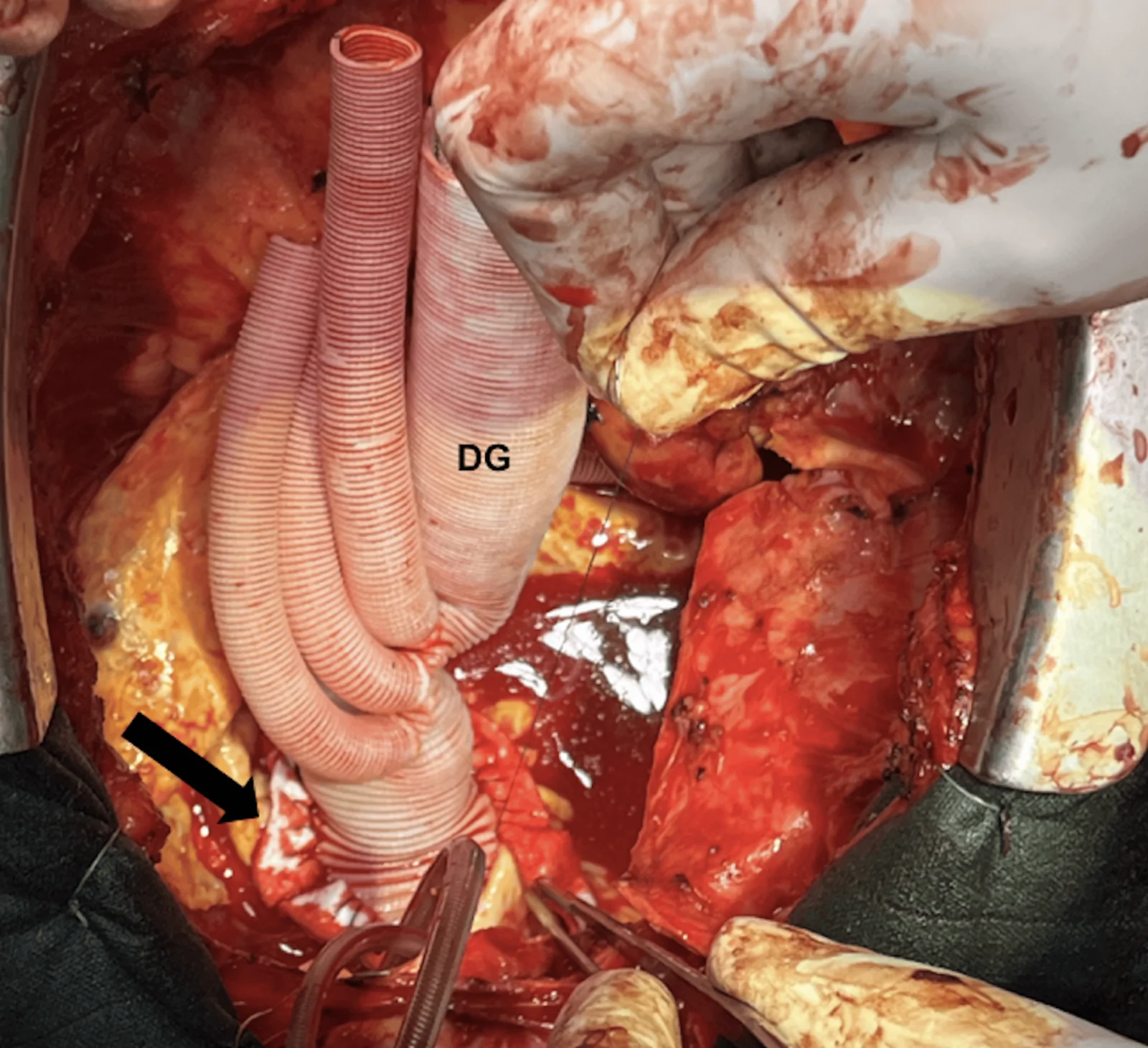
Distal arch reconstruction under deep hypothermic circulatory arrest Intraoperative view showing distal anastomosis of the quadrifurcated Dacron graft (DG) to the aortic arch during deep hypothermic circulatory arrest. The black arrow indicates the distal suture line reinforced with Teflon felt.

Systemic cooling was performed to a target temperature of 22°C using an α-stat blood gas management strategy. At 22°C, circulatory arrest with selective antegrade cerebral perfusion (SACP) was instituted through the brachiocephalic trunk and left common carotid artery. Aortic root and total arch replacement was performed using a 24-mm four-branch Gelweave™ Plexus graft (Terumo Aortic, Inchinnan, Scotland, UK). The distal anastomosis was reinforced with Teflon felt and sealed with BioGlue® (CryoLife Inc., Kennesaw, GA, USA), after which cardiopulmonary bypass flow was re-established through the fourth branch of the graft (Figure 2). 

**Figure 3:**
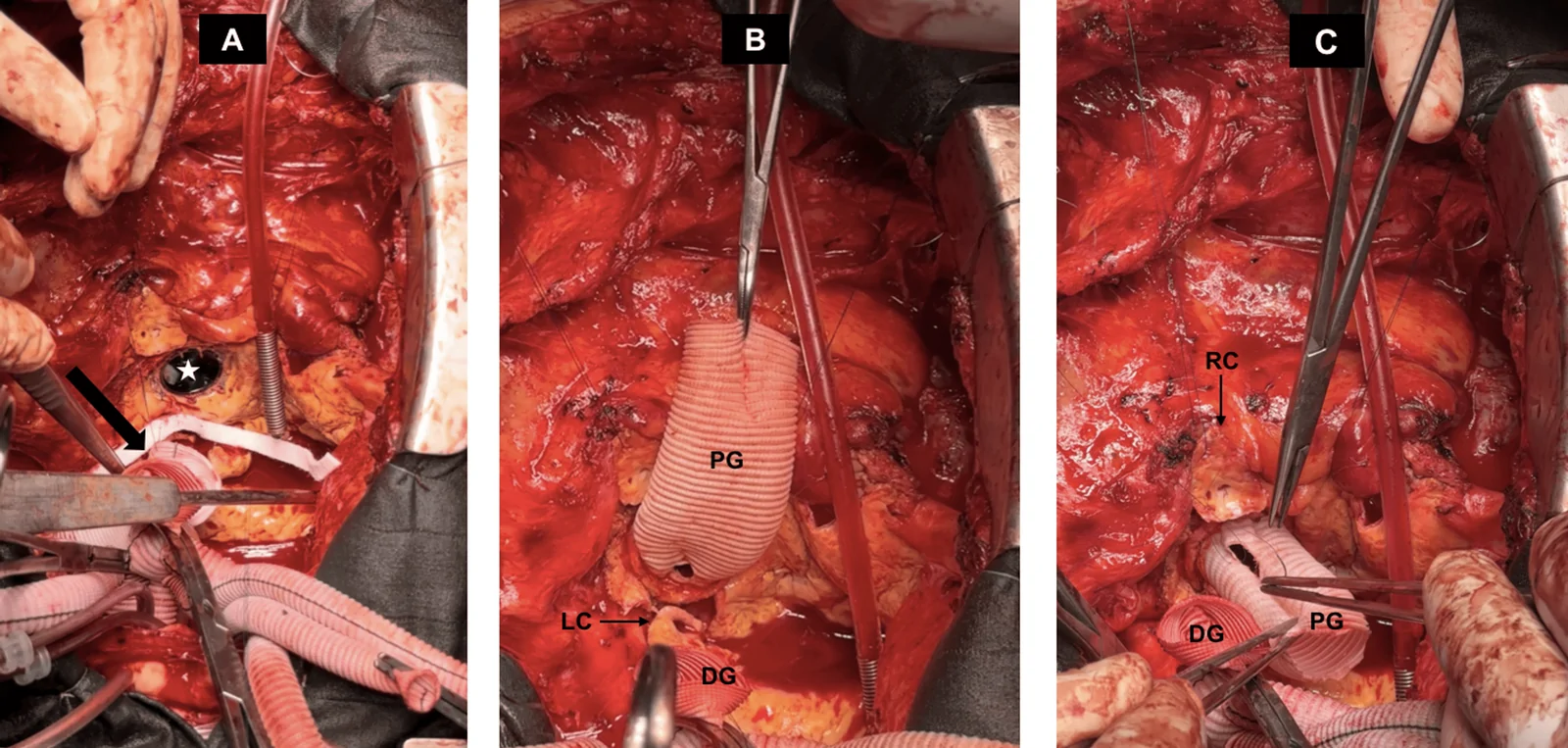
Sequential steps of proximal reconstruction following distal arch anastomosis (A) Aortic root exposure showing the mechanical aortic prosthesis (white asterisk) and the proximal graft reinforced with Teflon felt (black arrow). (B) Proximal graft (PG) positioned for left coronary button reimplantation. (C) Completed proximal reconstruction following coronary reimplantation. LC, left coronary button; DG, distal graft; RC, right coronary button; PG, proximal graft; DG, distal graft

The proximal anastomosis was then completed with reimplantation of both coronary buttons, followed by end-to-end reconstruction between the proximal and distal graft segments, allowing removal of the aortic cross-clamp and myocardial reperfusion (Figure 3). 

**Figure 4:**
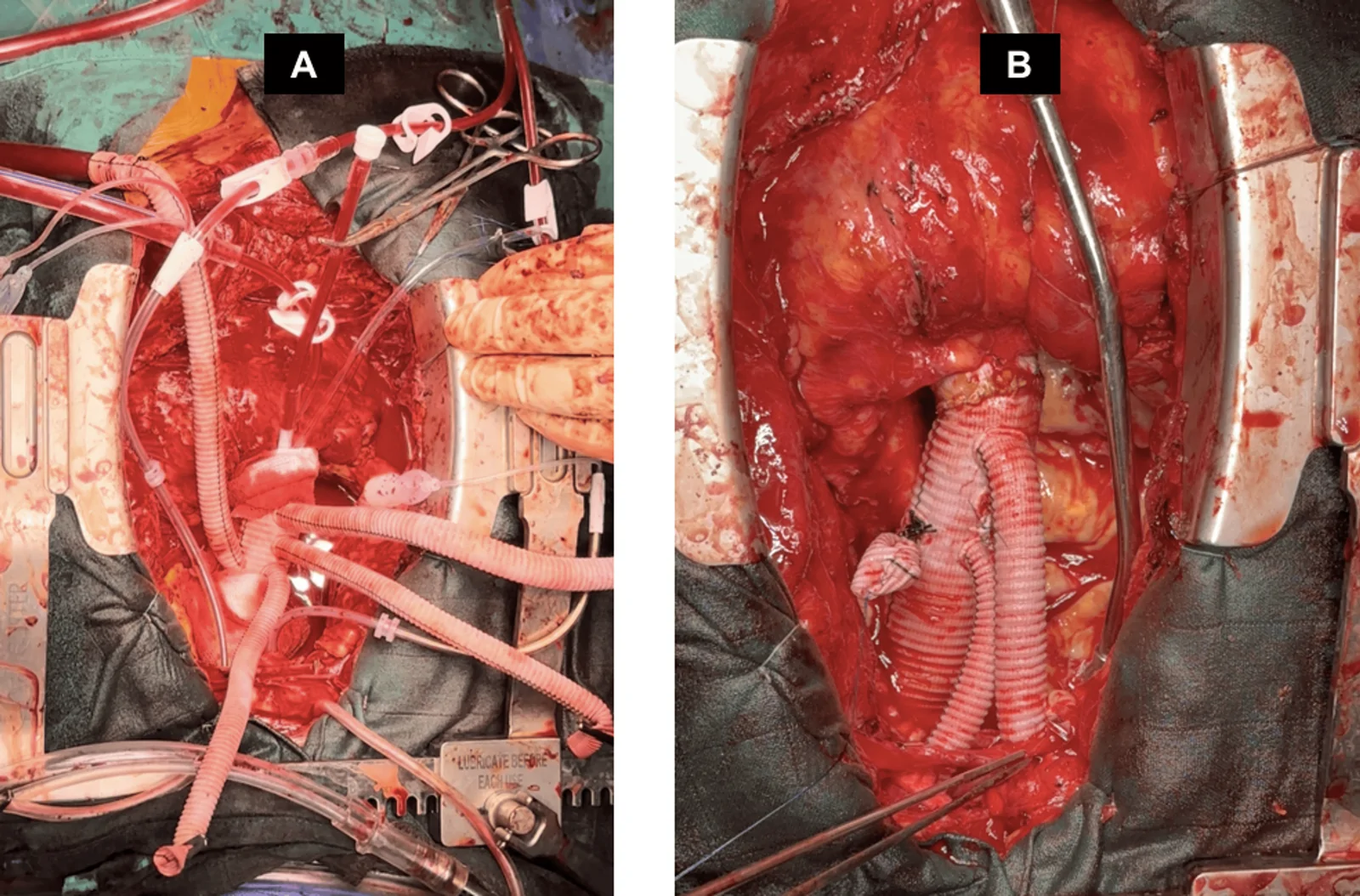
Supra-aortic trunk reimplantation and final operative reconstruction (A) Quadrifurcated Dacron graft positioned for sequential reimplantation of the supra-aortic trunks during arch reconstruction. (B) Final operative view following completion of supra-aortic trunk reimplantation, demonstrating restoration of continuity between the ascending aorta and aortic arch.

Finally, the supra-aortic trunks were reimplanted sequentially, beginning with the left common carotid artery and followed by the brachiocephalic trunk (Figure 4). 

The total cardiopulmonary bypass time was 439 minutes, aortic cross-clamp time was 110 minutes, and circulatory arrest time was 72 minutes under deep hypothermia. Inotropic support consisted of dobutamine (5 µg/kg/min) and norepinephrine (0.4 µg/kg/min). Transesophageal echocardiography following rewarming confirmed preserved biventricular function and normally functioning prosthetic valves.

The early postoperative course was complicated by mediastinal bleeding requiring surgical re-exploration. The patient subsequently developed severe acute respiratory distress syndrome (ARDS), stage 3 acute kidney injury, cholestatic liver dysfunction, hypernatremia, and hypoalbuminemia, all of which resolved with multidisciplinary critical care management.

Postoperative transthoracic echocardiography demonstrated preserved left and right ventricular function, a mean transprosthetic aortic gradient of 10 mmHg, a mean transprosthetic mitral gradient of 4.8 mmHg, and moderate residual tricuspid regurgitation without hemodynamic consequences.

Postoperative CECT revealed a peri-prosthetic fluid collection measuring 61-62 mm surrounding the supra-aortic trunk reimplantation sites, without evidence of contrast extravasation or infection. The patient was discharged six weeks after surgery in stable condition with complete resolution of compressive symptoms.

At four-month follow-up, CECT demonstrated a stable peri-prosthetic collection measuring 65 × 55 mm without evidence of contrast extravasation, dissection, or pericardial effusion. As there were no clinical or radiological signs of infection, compression of adjacent structures, or hemodynamic compromise, conservative management with close clinical and radiological surveillance was continued, and no intervention was considered necessary.

## Discussion

Giant ascending aortic aneurysms are uncommon but may cause life-threatening complications, including rupture, dissection, and compression of mediastinal structures [1]. When they occur in patients with previous cardiac surgery, redo aortic root and total arch replacement becomes particularly challenging because adhesions, distorted mediastinal anatomy, and prosthetic valves substantially increase the risk of bleeding, cardiac injury, and myocardial ischemia during re-entry [2].

Careful preoperative planning is therefore essential. In patients with aneurysms closely adherent to the posterior sternum, establishing peripheral cardiopulmonary bypass before sternotomy allows controlled cardiac decompression and systemic cooling, reducing the risk of catastrophic injury during re-entry [3]. In the present case, femoro-femoral bypass was instituted before resternotomy, providing hemodynamic stability and facilitating safe dissection of the aneurysm and surrounding structures. This proactive approach contrasts with emergency cannulation following accidental rupture, a scenario associated with significant morbidity and mortality in redo arch surgery [4].

Left ventricular apical decompression represented a key component of the operative strategy. During systemic cooling, ventricular fibrillation is expected and may lead to progressive LV distension and retrograde pulmonary edema if adequate ventricular unloading is not established. Morales-Rey et al. highlighted the importance of individualized preoperative planning and early LV venting to facilitate safe re-entry in complex redo aortic surgery [5]. Similarly, Hohri et al. described a staged sternal opening strategy for sternal-adherent aneurysms after initiation of cardiopulmonary bypass [6], while Cohn reported direct transthoracic LV apical venting as an effective technique for ventricular decompression during challenging reoperative procedures [7]. In our patient, pre-sternotomy LV apical venting through a left anterolateral thoracotomy, combined with peripheral cardiopulmonary bypass and systemic cooling using an α-stat blood gas management strategy, provided continuous ventricular unloading and facilitated controlled dissection in this exceptionally high-risk redo arch procedure.

Myocardial and cerebral protection remain central concerns during aortic arch surgery. In this case, deep hypothermia (22°C) using an α-stat blood gas management strategy combined with selective antegrade cerebral perfusion through the brachiocephalic trunk and left common carotid artery provided cerebral protection during circulatory arrest. Contemporary neuroprotection strategies combine varying degrees of hypothermia with antegrade cerebral perfusion, although no single approach has demonstrated clear superiority across all clinical settings [8]. Recent evidence suggests that mild hypothermic circulatory arrest combined with selective antegrade cerebral perfusion may achieve satisfactory short-term outcomes while reducing operative duration and perioperative morbidity [9]. Nevertheless, in complex redo arch procedures where prolonged dissection and extended circulatory arrest may be anticipated, deeper levels of hypothermia remain a reasonable strategy for additional organ protection.

The postoperative course was marked by bleeding requiring surgical re-exploration, respiratory failure, acute kidney injury, and transient multiorgan dysfunction. Such complications are well recognized after complex redo aortic surgery because prolonged cardiopulmonary bypass, extensive dissection, and tissue fragility increase the risk of postoperative morbidity. In a 10-year experience of redo total arch replacement, Zhao et al. reported an in-hospital mortality of 6.8% and a major adverse event rate of 13.7% [4]. Similarly, Ram et al. demonstrated that reoperative arch replacement carries significantly greater perioperative risk than primary procedures, although acceptable long-term survival can be achieved in experienced centers [10]. In our patient, prompt management of postoperative complications and coordinated multidisciplinary care resulted in complete clinical recovery despite a prolonged and complex postoperative course. 

As a single-case experience, the findings cannot be generalized; however, the report highlights a reproducible operative strategy that may be considered in selected patients with giant sternal-adherent aneurysms requiring complex redo arch surgery.

## Conclusions

Redo aortic root and total arch replacement after previous double mechanical valve replacement remains a formidable surgical challenge. This case highlights the importance of individualized preoperative planning and tailored cannulation strategies to facilitate safe re-entry while ensuring adequate cerebral and systemic organ perfusion in the event of catastrophic injury. In selected high-risk patients with giant sternal-adherent aneurysms, a staged reoperative strategy incorporating femoro-femoral cardiopulmonary bypass, pre-sternotomy LV apical decompression, and selective antegrade cerebral perfusion may contribute to favorable surgical outcomes.
